# Effortful Control and Community Violence Exposure as Predictors of Developmental Trajectories of Self-serving Cognitive Distortions in Adolescence: A Growth Mixture Modeling Approach

**DOI:** 10.1007/s10964-020-01306-x

**Published:** 2020-08-25

**Authors:** Concetta Esposito, Gaetana Affuso, Mirella Dragone, Dario Bacchini

**Affiliations:** 1 Department of Humanistic Studies, University of Naples “Federico II”, Napoli, Italy; 2 Department of Psychology, University of Campania “Luigi Vanvitelli”, Caserta, Italy

**Keywords:** Adolescence, Community violence exposure, Developmental trajectories, Effortful control, Self-serving cognitive distortions

## Abstract

Self-serving cognitive distortions are biased or rationalizing beliefs and thoughts that originate from the individual persistence into immature moral judgment stages during adolescence and adulthood, increasing the individual’s engagement in antisocial or immoral conducts. To date, the literature examining trajectories of cognitive distortions over time and their precursors is limited. This study sought to fill this gap, by examining effortful control and community violence exposure as individual and environmental precursors to developmental trajectories of cognitive distortions in adolescence. The sample consisted of 803 Italian high school students (349 males; *M*_age_ = 14.19, SD = 0.57). Three trajectories of cognitive distortions were identified: (1) moderately high and stable cognitive distortions (*N* = 311), (2) moderate and decreasing cognitive distortions (*N* = 363), and (3) low and decreasing cognitive distortions (*N* = 129). Both low effortful control and high exposure to community violence were significant predictors for moderately high and stable trajectory of cognitive distortions. These results point to the importance of considering moral development as a process involving multiple levels of individual ecology, highlighting the need to further explore how dispositional and environmental factors might undermine developmental processes of morality.

## Introduction

Social-cognitive theories posit that people act upon their interpretation of social events (Crick and Dodge 1994). According to Bandura’s (1986) social-cognitive formulations, the cognitive evaluation of events that take place in the child’s environment, how the child interprets these events, and how competent the child feels in responding in various ways provide the basis for child’s purposeful action. Growing up in a violent environment may lead children to see the world as a hostile and dangerous place (Guerra et al. 2003; Schwartz et al. 2000), and that violence itself is a useful means for conflict resolution (Dodge et al. 2006). The internalization of these schemas of the world, along with the development of normative beliefs about violence, amplify the risk for behaving aggressively. In terms of such cognitive process models, self-serving cognitive distortions well represent schemas that influence the individual’s encoding, interpretation, attribution, and evaluation, and thereby, impact the individual’s behavior in social situations (Barriga et al. 2001). However, to date, few studies have systematically examined the precursors of biases affecting moral cognition (Dragone et al. 2020; Hyde et al. 2010). Nonetheless, it is noteworthy that the processing of information for behaving in a specific way is a function of one’s ability to assemble action-relevant information that can be used to guide action. This capability supports the exhibition of self-control by directing attention, delaying or suppressing inappropriate responses, and moderating emotional arousal (Wikström and Treiber 2009). Overall, a number of studies have suggested a key-role of self-regulatory abilities in the development of moral emotions and behavior (Eisenberg et al. 2016), whereas studies examining possible associations between self-regulation and moral cognition are scarce. Starting from these considerations and using a social-cognitive perspective, the current investigation is intended to fill the gap in the literature by investigating the role of exposure to community violence and self-regulatory abilities—representing risk factors at environmental and individual levels of the individual’s ecology—in predicting specific longitudinal trajectories of moral cognitive distortions.

### Cognitive Distortions in the Framework of Moral Developmental Delay

The notion of self-serving cognitive distortions was introduced by Gibbs et al. to describe young offenders’ inaccurate or biased ways of attending to or conferring meaning upon experiences (e.g., Barriga and Gibbs 1996; Gibbs et al. 1995). Such cognitive errors were defined as “self-serving” as they assist the individual to self-justify acts that are in contrast with moral beliefs, such as antisocial behaviors, thus protecting the self from developing a negative self-image. Following Gibbs’ (2013) theory of moral development, self-serving cognitive distortions originate from a delay that occurs in moral development.

There are several aspects of Gibbs’ approach to the study of morality that are common to other current developmentalist theories (e.g., social domain theory; Smetana 2006; Turiel 1998), such as the importance of social experiences in constructing children and adolescents moral judgments (Arsenio and Lemerise 2004; Dodge and Rabiner 2004). However, differently from other moral theorists that emphasize the role of the social context, Gibbs retains the Kohlberg’s cognitive-developmental perspective (Kohlberg 1984), hypothesizing four stages of moral development, grouped into immature and mature levels (2013). This perspective based on developmental stages has been supported in reviews of studies conducted in over 40 countries around the world (Gibbs 2013), specifically showing an age trend of moral decision-making from immature to mature stages. Starting from a re-conceptualization of Kohlberg’s (1984) moral developmental theory, Gibbs assumes that the sequence of developmental stages identified by Kohlberg is not an obligatory process, and a moral delay can happen, with the persistence of the earliest levels of morality in adolescence and adulthood. As reported by Gibbs (2013), in this stage of arrested moral development, adolescents display cognitive biases (i.e., cognitive distortions), making her/him judge their moral transgression as acceptable and does not feel the deviant behavior as dissonant with common moral standards. Organizing the extant literature on cognitive distortions, Gibbs et al. (1995) introduced a four-category typological model of self-serving cognitive distortions: self-centered, blaming others, minimizing/mislabeling, and assuming the worst. “Self-Centered” cognitive distortions are defined as attitudes wherein the individual focuses on his/her own opinions, expectations, needs, and rights to such an extent that the opinions or needs of others are rarely considered or respected. “Blaming Others” involves cognitive schemas of misattributing the blame for one’s own behavior to sources outside the individual (i.e., external locus of control). “Minimizing” is defined as distortions, where the antisocial behavior is seen as an acceptable, perhaps necessary, way to achieve certain goals. “Mislabeling” is defined as a belittling and dehumanizing way of referring to others. Finally, “Assuming the Worst” represents cognitive distortions where the individual attributes hostile intentions to others, considers the worst-case scenario as inevitable, or sees his/her own behavior as beyond improvement.

In literature, there are several other theoretical accounts of self-serving cognitive distortions (e.g., Bandura’s moral disengagement, or Sykes’ and Matza’s neutralization theory), that, however, do not overlap with Gibbs et al.’s (1995) work. Specifically, a first consideration one might give in comparing Bandura’s moral disengagement perspective with Gibbs et al.’s self-serving cognitive distortions concerns the moral functioning they hypothesized as being behind the cognitive mechanisms that lead to behave (or not behave) aggressively. Indeed, according to Bandura, moral (or immoral) agency depends on self-regulatory processes of individual moral standards and anticipatory self-sanctions that must be activated to come into play (Bandura 2002), rather than depending on moral reasoning. As a result, selective activation and disengagement of internal control would permit various types of conduct with the same moral standards. Self-serving cognitive distortions, as theorized by Gibbs et al. (1995), instead, seem to be relatively stable cognitive mechanisms that, once internalized, are applied by the individual in the interaction within the social environment. Furthermore, the conceptualization of the relationship between cognitive distortions and antisocial or aggressive behavior is thought to be quite different across models. While other theories posit that they precede (or occur during) antisocial behavior, Gibbs et al. (1995) suggest the possibility of multidirectional causality, so that self-serving cognitive distortions may precede and/or follow behavior. In their attempt to integrate various neutralization concepts in a unique moral neutralization approach, Ribeaud and Eisner (2010) noticed that self-serving cognitive distortions, as conceptualized by Gibbs et al., did not totally overlap with Sykes and Matza (1957) or Bandura’s (2002) moral disengagement, in that they are theorized to more specifically conflate moral rationalizations with biased information processing. Assuming the worst, in particular, which partly overlaps Bandura’s concept of external attribution of blame, overcomes such a concept, including the attribution of hostile intentions to others, that is typical of social information processing models (Crick and Dodge 1994).

### Effortful Control and Community Violence as Precursors of Trajectories of Cognitive Distortions

According to the social-cognitive perspective, there are several individual and contextual factors that may contribute to the emergence of certain specific cognitive routines, scripts, and schemas (Huesmann 1998). In the stadial framework of moral cognitive development theorized by Gibbs, cognitive distortions are supposed to decline with maturation of the prefrontal cortex, thus suggesting that self-regulatory abilities, that are related to the functioning of the prefrontal cortex, are required for the maturation of moral decisions (Gibbs 2013). However, studies that investigate moral cognition together with self-regulatory abilities are not common in the literature. Overall, self-regulation has been studied by developmental researchers based on a temperamental perspective. Research in the moral field has primarily investigated the role of self-regulatory abilities in the development of moral emotions and behavior (Eisenberg et al. 2016), with a specific focus on the role of effortful control. Rothbart et al. defined effortful control as the self-regulation component of temperament, pertaining to the ability of voluntary inhibiting behavior when appropriate (called “inhibitory control”), activating behavior when needed (called “activation control”), and/or focus or shift attention as needed (called “attentional control”) (Rothbart et al. 2011). Empirical studies have showed that effortful control is related to adolescent sympathy and prosocial behaviors (Carlo et al. 2012; Eisenberg 2010). Indeed, effortful control would enable children to modulate emotional responses adequately to focus on the needs of others (experiencing sympathy) and assist others (acting in morally desirable ways with others). To our knowledge, little is known about whether and how effortful control is associated with the development of moral cognition, although there is empirical evidence supporting the hypothesis that they could be related. First, both low effortful control and bias in cognitive distortions have been found to predict aggressive adolescent behavior (e.g., Esposito et al. 2017; Hardy et al. 2015). Furthermore, it is hypothesized in social-cognitive models, that children who are poor at regulating emotions or lacking in focused attention are more likely to fail with information processing and show biases in the evaluation of social events (Wikström and Treiber 2009). However, to date, these relationships have not been examined in a systematic way.

By contrast, how context may influence the acquisition of pro-violent attitudes is well explained by social-cognitive theories, such as the observational learning theory (Bandura 1978). What children learn from models they observe within their daily contexts are not only specific behaviors, but also complex social scripts that, once established because of repeated exposure, are easily retrieved from memory to serve as cognitive guides for behavior (Dodge et al. 2006). Through inferences they make from repeated observations, children also develop beliefs about the world in general and about what kind of behavior is acceptable. Overall, it is theorized that being exposed to violent contexts increases the likelihood that children will (1) incorporate aggressive social scripts, (2) develop hostile and unsafe world schemas for interpreting environmental cues and making attributions about others’ intentions, and (3) acquire normative beliefs about violence, suggesting the appropriateness of behaving aggressively (Huesmann 1998). This process results in more sanctioning violent beliefs, more positive moral evaluations of aggressive acts, and in more justification for inappropriate behavior, inconsistent with society’s and individual’s moral norms. This is defined by Huesmann and Kirwil (2007) as “cognitive desensitization to violence.” Indeed, although desensitization is more properly used to refer to emotional changes that occur with repeated violence exposure, it is possible to also talk about “desensitization” when changes regard cognitive aspects that lead the individual to develop stronger pro-violent attitudes (i.e., attitudes approving violence as a means of regulating interpersonal contacts; Huesmann 1998). Consistent with this idea, several years before, Ng-Mak et al. (2002) formulated the “pathologic adaptation” model identifying repeated exposure to community violence as a precursor to a normalization of violence through moral disengagement, in which youths then experience less emotional arousal in response to violence, on one hand, and facilitates aggressive behavior, on the other. However, the development of moral disengagement as a consequence of repeated exposure to violence has not yet been tested empirically in a systematic way.

Beginning with the idea that potential precursors of moral disengagement should be experiences that directly model or at least expose children to attitudes and beliefs condoning the use of antisocial behavior (e.g., distribution and selling of illegal drugs, using violence as a primary conflict resolution strategy), Hyde et al. (2010) focused on neighborhood impoverishment as a potential precursor of moral disengagement, finding a significant association. Moreover, Wilkinson and Carr (2008) tried to raise this point using qualitative data from male violent offenders, reporting that individuals respond to exposure to violence in many ways, some of which seemed to be consistent with traditional concepts of moral disengagement.

Nonetheless, several studies have found significant associations between community violence and acceptance of violence cognitions, or bias of social information processing (see, for example, Allwood and Bell 2008; Bradshaw et al. 2009), whereas Bacchini et al. (2013) showed that higher levels of exposure to community violence as a witness, along with the perception of higher levels of deviancy among peers, reduced the strength of moral criteria for judging moral violations. Overall, to the best of our knowledge, only one study (Dragone et al. 2020) has systematically examined how being exposed to community violence is associated with cognitive distortions as intended in their moral dimension (Arsenio and Lemerise 2004; Ribeaud and Eisner 2010), finding a significant, even marginal, association of witnessing community violence with cognitive distortions over time. Furthermore, this relationship was not bidirectional (i.e., cognitive distortions did not predict violence exposure over time), shedding light on the importance of further investigate the predictive relationship of violence experiences within the community on the development of cognitive distortions over time.

## The Present Study

The central goal of this study was to investigate how low effortful control and exposure to community violence were associated with developmental trajectories of moral cognitive distortions. As this was the first study focusing on self-serving cognitive distortions as theorized by Gibbs, no specific hypotheses were done about developmental trajectories. Overall, it was expected that the membership to the most “distorted” group (e.g., high levels of cognitive distortions that show stability or increment over time) was predicted by low effortful control and high frequency of exposure to community violence. Adolescent gender and social desirability were included as controls to ensure that the associations of effortful control and violence exposure with trajectories of CDs were adjusted for their potential confounding effects.

## Methods

### Participants and Procedure

The participants were part of a currently ongoing longitudinal study (ALP; Arzano Longitudinal Project) aimed at investigating the determinants and pathways of typical and atypical development from early to late adolescence. The study design included four data points (one-year intervals) from two cohorts of adolescents who were enrolled in grade nine (Time 1 of the study) in 2013 and 2016, respectively. At Time 1 (T1), the sample consisted of 803 Italian adolescents (349 males and 454 females; *M*_age_ = 14.19, SD = 0.57) attending several public schools in the metropolitan area of Naples (Italy). The most part of the sample was recruited from two high schools (34.4% and 41.9%), whereas the remaining proportion came from other 20 schools in the same geographic area. The neighborhoods served by these schools is characterized by serious social problems, such as high unemployment (41%), high school-dropout rates (18.8%), and the presence of organized crime, with rates that are among the highest in Italy [Istituto Nazionale di Statistica (ISTAT) 2016]. National statistics are also supported by findings of prior empirical research, documenting that adolescents living in Naples are massively exposed to neighborhood violence in their everyday life (Bacchini and Esposito 2020).

Data collection took place during the spring of 2013 and 2016 (T1), 2014 and 2017 (Time 2; T2), 2015 and 2018 (Time 3; T3) and 2016 and 2019 (Time 4; T4). Parents’ written consent and adolescents’ assent were obtained prior to the administration of questionnaires, which was conducted during classroom sessions by trained assistants. Additionally, they were informed about the voluntary nature of participation and their right to discontinue at any point without penalty. The socioeconomic condition of participants’ families reflected the Italian National profile (Istituto Nazionale di Statistica (ISTAT) 2016). Approximately 50% of the fathers and mothers had a low level of education (middle school diploma or less), 28% had a high school diploma and approximately 8% had a university degree.

### Measures

#### Effortful control

To evaluate temperamental effortful control at T1, adolescents were asked to rate items from the long version of the Early Adolescent Temperament Questionnaire—Revision (EATQ-R) (Ellis and Rothbart 2001). Items were rated on a 5-point Likert scale ranging from, “*almost never true*” (1) to “*almost always true*” (5). The multi-componential structure of effortful control has arisen from the factorial analysis of the questionnaire (Ellis and Rothbart 2001). The measure was computed by averaging item ratings of the activation control (e.g., “If I have a hard assignment to do, I get started right away”), attention control (e.g., “I pay close attention when someone tells me how to do something”), and inhibitory control (e.g., “When someone tells me to stop doing something, it is easy for me to stop”) scales, after recoding inversely formulated items (*α* = 0.77, *ωh* = 0.67). Items were translated from English into Italian by two native Italian speakers, experts in psychology and fluent in English, and then back translated by a native English speaker to ensure its comparability to the English version.

#### Exposure to community violence

Exposure to community violence was self-reported during T1 using the Exposure to Community Violence Questionnaire (Esposito et al. 2017), consisting of 12 items. Adolescents were asked to report the frequency (from 1 = “*never”* to 5 = “*more than five times”*) of being a victim or a witness of violent incidents that had occurred during the last year in their neighborhood. Sample items were, “How many times have you been chased by gangs, other kids, or adults?” and “How many times have you seen somebody get robbed?” For each participant, items of each scale were averaged to form a global score of exposure to community violence (*α* = 0.85, ω*h* = 0.68).

#### Self-serving cognitive distortions

At each time point, participants responded to the 39 items in the, “How I think Questionnaire” (HIT) (Barriga et al. 2001; Italian validation by Bacchini et al. (2016)), measuring self-serving cognitive distortions. Each item was rated on a 6-point Likert scale from, “*agree strongly*” to “*disagree strongly*.” A sample item was: “If someone leaves a car unlocked, they are asking to have it stolen.” The mean response to the 39 items is the overall HIT score, with higher scores indicating higher levels of cognitive distortions (*αs* range from 0.95 to .097; *ωh* range from 0.84 and 0.90).

#### Social desirability

As a control variable, participants were asked to complete 12 items from the Lie scale of the Big Five Questionnaire (Caprara et al. 1993) to asses social desirability bias. Items were rated on a 5-point Likert-type scale ranging from “*very false for me*” to “*very true for me*”. Sample items were: “I’ve always gotten along with everyone” and “I’ve never told a lie.” The scale score was created by averaging items score, with higher scores reflecting higher levels of socially desirable responding (*α* = 0.77; *ωh* = 0.78).

### Attrition Analysis

All T1 participants completed the HIT Questionnaire at T2. Seventy-two (9%) of T1 and T2 participants did not complete the questionnaire at T3, and 151 (18.8%) at T4. The total attrition rate was mainly due to the absence of adolescents from school during assessments. The Little’s test (Little and Rubin 2002) for data was missing completely at random (MCAR) in SPSS 21 was significant, *χ*^2^ (26) = 73.69, *p* < 0.001, suggesting that missingness was not completely at random. Independent *t*-tests comparing mean differences between missing and non-missing cases suggested that those who dropout on measures of cognitive distortions at T3 and T4 reported higher levels of cognitive distortions at previous time point, and higher levels of community violence exposure, effortful control and social desirability at T1 (all *ps* < 0.05).

### Analytic Approach

The main analyses were conducted in Mplus 8 (Muthén and Muthén 1998-2017). Missing data were handled using full-information maximum-likelihood (FIML) method with the assumption that the data were missing at random (MAR; Little and Rubin 1989). As indicated in previous work (Wang and Bodner 2007), FIML is an especially useful missing-data treatment in longitudinal designs because the outcome scores for dropouts tend to be correlated with their own previously recorded responses from earlier waves (i.e., an MAR pattern).

Latent growth mixture models (GMMs) were used for identifying distinct growth developmental trajectories (or classes) of cognitive distortions (Muthén 2004; Muthén and Muthén 2000). Extending the logic of multiple-group growth models, where groups are defined a priori, the GMM identifies classes of individuals post hoc, such that individuals that are in the same class have similar trajectories and individuals in different classes have sufficiently divergent trajectories (Grimm et al. 2017). Using a person-centered approach, GMMs are extremely useful for developmental researchers, in that they allow not only the identification of different classes of intra-individual (within-person) change, but also to test hypotheses about inter-individual (between-person) differences in that intra-individual change by examining (1) antecedents, or predictors, and (2) consequences, or distal outcomes, of class membership (Wickrama et al. 2016).

The GMM analysis consisted of three steps. First, simple growth models were run to determine the growth parameters for the GMM. Specifically, three unconditional (without any covariate) latent growth models were tested and compared: (1) intercept only, in which each individual has an intercept, but no change over time is estimated, (2) intercept and linear slope, which allows individual scores to change linearly over time and permits individuals to differ in their rates of change, and (3) intercept and quadratic slope, in which the rate of change is assumed to be non-linear. To compare these nested models, the value of −2log likelihood (−2LL) was used to perform *χ*^2^ tests with degrees of freedom equal to the difference in the number of degrees of freedom between the models.

Second, once the best model was tested for the linear growth model, five specific models were fit to examine class differences within certain parameters of the linear growth model. The first model (M1; Table 2) was the baseline (invariance) model, where all estimated parameters were invariant across classes. This model treats the data as if there is only one class. The second model (M2; Table 3) was a latent class growth model (LCG), in which means are estimated and within-class variances fixed to zero. This model assumes that all individual trajectories within a class are homogeneous. In the third model (M3; Table 3), the means of the intercept and slope are class specific. That is, individuals are probabilistically placed in classes that differ in their baseline levels and rates of change.

The fourth model (M4; Table 3) is the means and covariances model, where the average trajectories, the magnitude of between-person differences in the intercept and slope, and the association between intercepts and slopes within each class are class specific. Finally, in the fifth model (M5; Table 3), classes are allowed to differ in all estimated parameters of the linear growth model: means, covariances, and residual variances. These latter, particularly, provide information about within-person fluctuations in scores over time. As recommended by Grimm et al. (2017), within each model type, models with various numbers of classes were fit, starting with two-class models, and increased the number of latent classes incrementally until the model encountered convergence issues or the model fit indicates that additional classes are unlikely to produce viable results.

Models were compared based on fit criteria and the interpretation of model parameters. First, model convergence was examined. Second, it was examined the information criteria: BIC, AIC, and sample size adjusted BIC. In general, lower values indicate a better fitting model. Third, likelihood ratio tests [Lo Mendell Rubin (LMR-LRT), or the bootstrap likelihood ratio test], which provide additional information for model selection within each model type (e.g., M2 models), were examined. Statistically significant values indicate that dropping one class from the model would significantly worsen the model fit. Afterward, a probability lower than 0.001 for a two-class model indicate that this is preferred to the one-class model, and so on.

Fourth, it was examined the entropy statistic and the average posterior probabilities. Specifically, entropy is a standardized index (i.e., ranging from 0 to 1) of model-based classification accuracy. Higher values indicate improved enumeration accuracy, which indicates clear class separation (Nagin 2005). The entropy statistic is based on estimated posterior probabilities for each class. For example, a probability of 0.91 suggests that 91% of subjects in the assigned class fit that category, while 9% of the subjects in that given class are not accurately described by that category (Fanti and Henrich 2010). Overall, the model with lower information criteria, higher entropy, average posterior probability values, and statistically significant *p* values for the likelihood ratio tests show the better fit. However, this information was also supplemented with substantive knowledge of the phenomena being studied to identify the model that best represented the data, as generally recommended (Grimm et al. 2017; Muthén 2003).

After selecting the optimal model, the third and final step was to extend the GMM to include covariates as predictors of trajectory class membership. Some researchers (Li and Hser 2011; Lubke and Muthén 2007; Muthén 2004) have recommended using the model with covariates when determining the appropriate number of latent classes, whereas, others (Enders and Tofighi 2007) have argued that class enumeration should be done without covariates. The latter approach was used, because it assures that the latent classes are based on the longitudinal trajectories and not the covariates (Grimm et al. 2017).

**Fig. 1:**
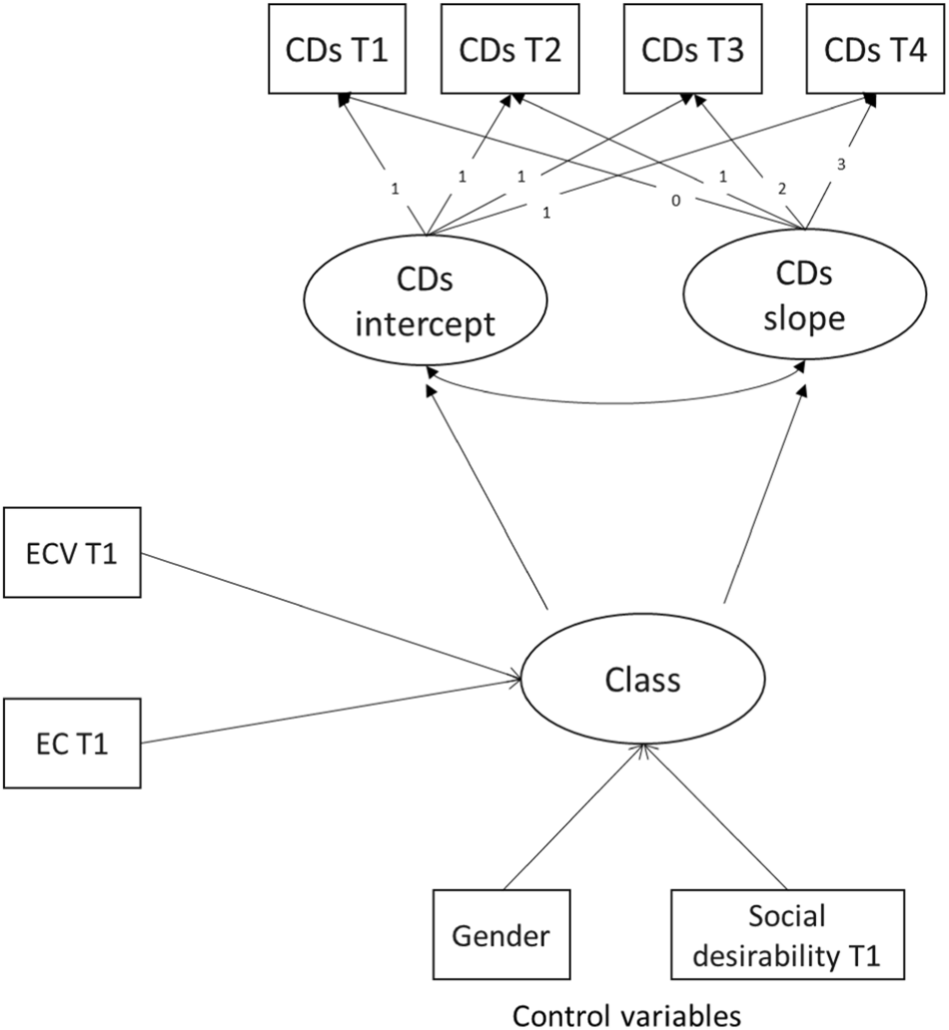
Path diagram of the hypothesized three-step GMM. CDs self-serving cognitive distortions; EC Effortful control; ECV Exposure to community violence

Following this suggestion, auxiliary variables were included as predictors in the GMM using a three-step approach (Nylund-Gibson et al. 2014). After the first step, which consisted in the estimation of the unconditional (without covariates) mixture model, in the second step, individual probabilities of trajectory class membership estimated from the latent class posterior probabilities were used to classify individuals into one or another class, while retaining knowledge of the uncertainty of that classification (in principle, the standard error of that classification). Third, a new latent GMM was formulated that examined the relationships among the covariates and the latent class variable. Thus, the effects of covariates can be studied while both assuring that the latent class variable is only derived from the repeated measures and the uncertainty inherent in the classification is taken into consideration. Specifically, adolescent gender, exposure to community violence and effortful control at T1 were added as predictors of longitudinal trajectories of cognitive distortions (see Fig. 1). According to this procedure, when a predictor is included in a GMM, a multinomial regression is performed to investigate the influence of a predictor on between-class variation.

## Results

Descriptive statistics and correlations between all variables used in the study are shown in Table 1. As can be observed, all the study’s variables significantly associated each other. Females reported marginally significant lower levels of community violence exposure and cognitive distortions at T2 and T3.

**Table 1:** Correlations, means and standard deviations (SDs)

	1	2	3	4	5	6	7	Range	Mean	SD
1. Gender (female)	1									
2. EC T1	−0.05	1						1–5	3.40	0.50
3. ECV T1	−0.08*	−0.26***	1					1–5	1.60	0.60
4. CDs T1	−0.03	−0.47***	0.33***	1				1–6	2.31	0.88
5. CDs T2	−0.11**	−0.45***	0.28***	0.61***	1			1–6	2.25	0.89
6. CDs T3	−0.16**	−0.41***	0.26***	0.45***	0.54***	1		1–6	2.22	0.94
7. CDs T4	−0.27***	−0.34***	0.26***	0.44***	0.46***	0.53***	1	1–6	2.03	0.92
8. Social desirability	−0.25***	0.30***	−0.06	−0.18***	−0.11**	−0.06	0.05	1–5	3.07	0.66

Gender was coded 1 = male and 2 = female

*EC* effortful control, *ECV* exposure to community violence, *CDs* self-serving cognitive distortions

*p < 0.05

**p < 0.01

***p < 0.001

The model fit information for all estimated models is presented in Table 2. The significant change of −2LL between the no-growth and linear growth models suggested that the linear growth model fit the data significantly better than the no-growth model. The change of −2LL was also marginally significant between the linear and quadratic growth models. However, the inspection of the other model fit indices and parameters indicated a better fit for the linear model.

**Table 2:** Fit indices and means of the intercept, linear, and quadratic slopes for the baseline model (one-class model)

		Baseline model (M1)	
	No-growth model	Linear growth model	Quadratic growth model
−2log likelihood	7054.41	6983.52	6973.51
AIC	7060.41	6995.52	6993.51
BIC	7074.48	7023.65	7040.40
RMSEA	0.11	0.07	0.10
Intercept mean	2.23***	2.32***	2.30***
Slope mean		−0.07***	0.00
Quadratic slope mean			−0.02
Δ −2log likelihood		70.89, *p* < 0.001	10.01, *p* = 0.04

***p < 0.001

When examining the model convergence of GMM, as noted by asterisks in Table 3, several models did not properly converge. Specifically, the variance of the slope was negative in one or more of the classes in the two- and three-class means models (M3) and covariance models (M4). In the four-class M4 and M5, the best log likelihood value was not replicated, indicating that additional classes are unlikely to produce viable results. Given these results, our evaluation was then specifically focused on M2 and M5.

**Table 3:** Model comparison

	Models									
Fit statistic	Latent class growth (M2)		Means (M3)		Means + covs (M4)			Means + covs + residual variances (M5)		
	2-class	3-class	2-class*	3-class*	2-class*	3-class*	4-class**	2-class	3-class	4-class**
Proportions	0.61/0.39	0.49/0.39/0.12	0.78/0.22	0.04/0.15/0.81	0.42/0.58	0.18/0.41/0.41		0.44/0.56	0.39/0.45/0.16	
AIC	7094.76	6950.61	6854.77	6802.64	6679.47	6618.19		6161.58	6001.21	
BIC	7122.89	6992.80	6896.96	6858.90	6735.73	6702.59		6222.53	6094.97	
ABIC	7103.83	6964.22	6868.38	6820.79	6697.63	6645.43		6181.25	6031.46	
Entropy	0.77	0.75	0.77	0.83	0.65	0.64		0.76	0.75	
VLMR *p* value	<0.001	0.05	<0.001	0.56	<0.001	0.007		<0.001	<0.001	
Bootstrap *p* value	<0.001	0.06	<0.001	0.57	<0.001	0.008		<0.001	<0.001	

*At least one non positive-definite covariance matrix

**Model did not converge

With respect to the information criteria (BIC, AIC, and sample size adjusted BIC), it was evident that there was a decrease when moving from one to two classes and from two to three classes in both models (M2 and M5). The likelihood ratio tests for M2 and M5 indicate that the two-class model was preferred to the one-class model (all *p*s < 0.001), and the three-class model was preferred to the two-class model (*p* = 0.05 and < 0.001, respectively). The entropy statistic was higher for the two-class LCG model (0.77) compared with the three-class LCG and two- and three-class means, covariances, and residual variance models. However, for the three-class models, the entropy value was the same for M2 and M5; diagonal probabilities for the three-class M5 ranged from 0.87 to 0.91, and from 0.82 to 0.91 in the three-class M2.

**Fig. 2:**
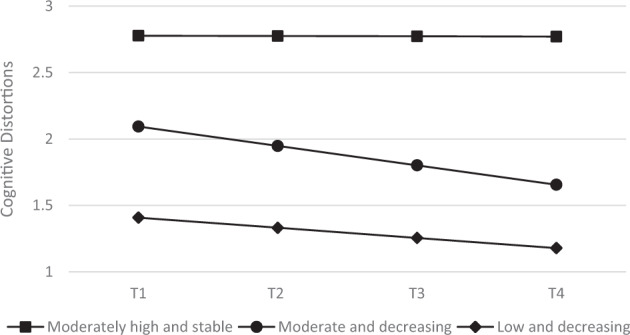
Trajectories of self-serving cognitive distortions. Three-class means, covariances and residual variances model

Based on this information, the three-class means, covariances, and residual variance model (M5) was selected as the best fitting model. The identified three trajectories classes are shown in Fig. 2: (1) moderately high and stable cognitive distortions (*N* = 311), (2) moderate and decreasing cognitive distortions (*N* = 363), and (3) low and decreasing cognitive distortions (*N* = 129).

Parameter estimates from this model are shown in Table 4. Specifically, the mean rate per year of cognitive distortions was not statistically significant for class 1. Class 2 and class 3 included adolescents with a significant negative mean rate of change over time. In class 1 and 2, there was a significant negative covariance between the intercept and slope, indicating that CD levels tend to decrease more slowly over time in adolescents who had higher values at T1. Moreover, there were significant between-person differences at the intercept in all classes, whereas significant between person differences in the slope were found for class 1 and 2.

**Table 4:** Parameter estimates for the chosen model: 3-class mean + covs + residual variances

	Class 1 (high and stable; *N* = 363)	Class 2 (moderate and decreasing; *N* = 311)	Class 3 (low and decreasing; *N* = 129)
Average probability of class membership	0.94	0.83	0.85
Latent variable means			
Intercept mean	2.78***	2.09***	1.41***
Slope mean	−0.002	−0.15***	−0.08***
Latent variable covariances			
Intercept variance	0.31***	0.16***	0.05*
Slope variance	0.05**	0.01*	0.00
Intercept-slope covariance	−0.09**	−0.03**	−0.01
Residual variance	0.64***	0.12***	0.02***

*p < 0.05

**p < 0.01

***p < 0.001

### Precursors of Developmental Trajectories of Cognitive Distortions

Table 5 presents the logistic coefficients and odds ratio resulting from the multinomial logit regression analysis, in which classes were regressed on exposure to community violence and effortful control at T1, controlling for gender and social desirability. In interpreting the multinomial coefficients, class 2 (moderate and decreasing) was used as the reference class. Specifically, the log-odds of being in class 1 (moderately high and stable) in comparison to the log-odds of being in class 2 increased with the amount of exposure to community violence. Similarly, low levels of effortful control and being male increase the log-odds of being in class 1 relative to class 2, whereas high effortful control increase the log-odds of being in class 3. No significant associations were found between social desirability and trajectories of class membership.

**Table 5:** The logit coefficients of predictors from the manual 3-step approaches

	Manual 3-step approach			
	Low and decreasing CDs^a^		High and stable CDs^a^	
Predictors	Est.	OR	Est.	OR
Male (vs. female)	0.20	1.22	−1.08***	2.94
ECV (T1)	0.24	1.27	1.38***	3.97
EC (T1)	1.92***	6.82	−2.65***	14.15
Social desirability	0.28	1.32	0.34	1.40

Unstandardized coefficients are shown

*ECV* exposure to community violence, *EC* effortful control, *Est*. estimate, *OR* odds ratio

^a^Medium and decreasing is the reference class

***p < 0.001

## Discussion

Self-serving cognitive distortions have been described as thinking errors that originate from the individual persistence into immature moral judgment stages during adolescence and adulthood, increasing the risk of individual’s engagement in antisocial or immoral conducts (Gibbs 2013). The aim of the current study was to investigate the trajectories of moral cognitive distortions in adolescence, and the simultaneous contribution of effortful control and community violence exposure as individual- and environmental-level factors, respectively, in making adolescents more vulnerable to use cognitive distortions when interpreting social situations. Using a person-centered approach, developmental trajectories of cognitive distortions in adolescence were first identified. Then, effortful control and being exposed to community violence were tested as potential predictors of those trajectories, controlling for adolescent gender and social desirability.

Overall, three trajectories were identified to best explain variation in cognitive distortions over time. Approximately 45% of the sample demonstrated a moderately high and relatively stable trajectory of cognitive distortions, whereas 39% reported initially moderate levels of cognitive distortions that decreased over time. Finally, a small number of participants (16%) showed low initial levels of cognitive distortions, with a decreasing tendency over time.

The identified trajectories were partially consistent with those identified by Paciello et al. (2008). The authors found a general decline of moral disengagement over time that, in their opinion, could reflect changes in cognitive and social structures occurring during adolescence and that, in turn, promote moral reasoning and then moral agency (Eisenberg 2000). Following Gibbs’ theory, the resulted declining trajectories of the current study, including about half size of the study’s sample, could be interpreted as a means of a developmental process, through which, along with other cognitive and socioemotional achievements, youths progress from a relatively superficial level, characterized by schema generating a higher use of egocentric and pragmatic self-serving thinking errors or cognitive distortions, to a more mature level of interpersonal and sociomoral reasoning, in which they can take on the roles or consider the perspective of others (Gibbs 1995). This perspective that recognizes that moral maturity occurs over time is also supported by research adopting the moral domain approach (Tisak and Turiel 1988) with children of different age. More specifically, moral domain theorists have showed that understanding within the moral domain develops from a focus on concrete harm in early childhood to an understanding of fairness in later childhood (Smetana 2006). Differently from cognitive-developmental theorist, however, the domain theory does not elaborate on the cognitive or affective processes that allow for this development to occur.

The other half size of the study’s sample was identified in the trajectory with initially moderately high and, over time, stable cognitive distortions. This finding highlights a high likelihood, for high school youths, to display a persistent and pronounced egocentric bias that consolidates into cognitive distortions (Barriga et al. 2000), perhaps reflecting a prolonged immature or superficial moral judgment stage. The condition where cognitive distortions persist over time has been described by Gibbs as a “moral developmental delay,” that has been widely documented among antisocial and delinquent youth (Gibbs 2013).

### Predictors of Cognitive Distortions Trajectories

When examining how effortful control and community violence exposure were related to developmental trajectories of cognitive distortions, the results indicated that both factors have an impact on the likelihood to show a developmental tendency rather than another. As concerns effortful control, two main results emerged: first, high effortful control was associated with initially lower and, over time, decreasing levels of cognitive distortions, supporting the hypothesis that moral development progresses along with cognitive and socioemotional development, as suggested by Gibbs (2013); and second, low effortful control predicted moderately high and stable cognitive distortions over time, indicating that the persistence of thinking errors during adolescence could depend on an individual failure to moderate emotional responses, direct attention, organize information, and delay impulsive action (Wikström and Treiber 2009).

Furthermore, it was found that a high frequency of exposure to community violence was a significant risk factor for being in the class with higher and tendentially stable cognitive distortions, relative to the moderate and decreasing class. This finding seems to be consistent with the hypothesis that youths could become desensitized to violence after repeated exposure (Huesmann and Kirwil 2007; Mrug et al. 2015), as well as with other previous findings highlighting a strict association between community violence and the development of positive attitudes toward violence (Allwood and Bell 2008), normative beliefs about violence (McMahon et al. 2013), or hostile attributional bias (Bradshaw et al. 2009). Despite the relatively great number of studies that have examined single indicators of the alteration of youths’ cognitive processes through their experiences of violence, the results of this study extend prior findings by addressing this issue from a moral perspective. This finding is supported by a recent study that investigated the longitudinal and reciprocal effects between community violence exposure and cognitive distortions in a sample of Italian adolescents (Dragone et al. 2020). More in detail, the authors found that violence exposure within the community significantly predicted cognitive distortions one year later, whereas cognitive distortions did not predict violence exposure longitudinally. What this approach allows for speculation is that growing up in a violent neighborhood, as well as aversive parenting or deviant peers in previous studies (Ribeaud and Eisner 2015), might undermine the normative process of moral development, thus, causing the moral delay hypothesized by Gibbs, that consolidates into self-serving cognitive distortions (Gibbs 2004).

Overall, these findings support the need for the design and implementation of preventive interventions aiming at strengthening youth moral cognition and enhancing individual self-regulatory abilities when facing with social problems. Examples of programs have been widely documented in the literature (Gibbs 2013; Steinberg 2014). Within Gibbs’ theoretical framework, a school-based program, the “EQUIP for educators,” has been developed and implemented in several contexts (Van der Velden et al. 2010). The EQUIP for educators is an adapted version of the original EQUIP program designed for the treatment of juvenile offenders (Equipping Youth to Help One Another; Gibbs et al. 1995). One of the most strength of this program, in line with this study’s findings, is that it takes into account and attempts to transform the distorted and harmful culture where cognitive distortions develop and persist over time. Through the establishment of a mutual help approach, this psychoeducational program aims to equip youth with mature moral judgment, and skills to manage anger and correct cognitive distortions.

With respect to control variables, males were more likely to show high and stable levels of cognitive distortions over time, whereas, gender was not a significant predictor of showing low and decreasing cognitive distortions. These results are consistent with gender differences found in the previous literature, such that, while moral judgment does not appear to vary according to gender and cognitive deficits do seem to represent risk factors for both genders (Barriga et al. 2001), males generally self-reported more cognitive distortions than females (Lardén et al. 2006; Owens et al. 2012). Further studies are yet needed to understand how these differences originate.

### Limitations and Future Directions

In interpreting these findings, some limitations must be considered. First, all measurements in the study relied exclusively on adolescent self-reporting. Despite the effects were controlled for social desirability in the current study, future studies may benefit from utilizing a multi-informant approach (e.g., parent reports for temperamental constructs) jointly with self-reporting measures. Furthermore, data concerning the frequency of being exposed to community violence could benefit from a more objective and comprehensive description of violence in the everyday lives of adolescents, including official data from national census agencies and police departments. Another limitation concerns the generalizability of the results, as the study included a sample from a limited geographic area in Southern Italy. As described above, the context where data were collected is characterized by serious social problems, that expose adolescents to high risk situations (e.g., violence exposure, youth delinquency, gang affiliation). This might shape culture-specific beliefs and values, that in turn might influence an individual’s cognitions and behaviors (Bacchini et al. 2015). More research is needed to confirm that the explanatory model proposed in this study applies to populations from other, possibly differing, cultural contexts. Furthermore, based on the current findings, future studies should investigate potential gender-related differences in pattern of development of cognitive distortions.

## Conclusion

The current findings extend the literature concerning the role of individual self-regulatory mechanisms and environmental factors on the development of moral cognition. Using a person-centered approach, the findings of the present study indicated that: (1) most adolescents exhibited declining levels of self-serving cognitive distortions over time, and (2) adolescents who exhibited initially higher levels of cognitive distortions also showed a tendency to remain stable over time; they were typically male youth, with low effortful control and a high frequency of being exposed to community violence. These results point to the importance of considering moral development as a process involving multiple levels of individual ecology. Future studies may deepen the investigation of other social, cognitive, and biological factors that could influence trajectories of moral cognition, both for expanding knowledge about the development and persistence of biases in moral cognition in adolescence (and their adjustment-related outcomes), and to design appropriate interventions that could prevent adolescents from developing these biases. The findings of the current study call specific attention to the need to support children and adolescents in developing skills that could help them to successfully cope with violence and high-risk environments, including self-regulatory abilities, social problem-solving skills and moral education.
